# Oxidative Stress and DNA Lesions: The Role of 8-Oxoguanine Lesions in *Trypanosoma cruzi* Cell Viability

**DOI:** 10.1371/journal.pntd.0002279

**Published:** 2013-06-13

**Authors:** Pedro H. N. Aguiar, Carolina Furtado, Bruno M. Repolês, Grazielle A. Ribeiro, Isabela C. Mendes, Eduardo F. Peloso, Fernanda R. Gadelha, Andrea M. Macedo, Glória R. Franco, Sérgio D. J. Pena, Santuza M. R. Teixeira, Leda Q. Vieira, Alessandra A. Guarneri, Luciana O. Andrade, Carlos R. Machado

**Affiliations:** 1 Departamento de Bioquímica e Imunologia, Instituto de Ciências Biológicas - UFMG, Belo Horizonte, Minas Gerais, Brazil; 2 Departamento de Bioquímica, Instituto de Biologia - UNICAMP, Campinas, Sa˜o Paulo, Brazil; 3 Centro de Pesquisas René Rachou - FIOCRUZ, Belo Horizonte, Minas Gerais, Brazil; 4 Departamento de Morfologia, Instituto de Ciências Biológicas - UFMG, Belo Horizonte, Minas Gerais, Brazil; McGill University, Canada

## Abstract

The main consequence of oxidative stress is the formation of DNA lesions, which can result in genomic instability and lead to cell death. Guanine is the base that is most susceptible to oxidation, due to its low redox potential, and 8-oxoguanine (8-oxoG) is the most common lesion. These characteristics make 8-oxoG a good cellular biomarker to indicate the extent of oxidative stress. If not repaired, 8-oxoG can pair with adenine and cause a G:C to T:A transversion. When 8-oxoG is inserted during DNA replication, it could generate double-strand breaks, which makes this lesion particularly deleterious. *Trypanosoma cruzi* needs to address various oxidative stress situations, such as the mammalian intracellular environment and the triatomine insect gut where it replicates. We focused on the MutT enzyme, which is responsible for removing 8-oxoG from the nucleotide pool. To investigate the importance of 8-oxoG during parasite infection of mammalian cells, we characterized the MutT gene in *T. cruzi* (TcMTH) and generated *T. cruzi* parasites heterologously expressing *Escherichia coli* MutT or overexpressing the TcMTH enzyme. In the epimastigote form, the recombinant and wild-type parasites displayed similar growth in normal conditions, but the MutT-expressing cells were more resistant to hydrogen peroxide treatment. The recombinant parasite also displayed significantly increased growth after 48 hours of infection in fibroblasts and macrophages when compared to wild-type cells, as well as increased parasitemia in Swiss mice. In addition, we demonstrated, using western blotting experiments, that MutT heterologous expression can influence the parasite antioxidant enzyme protein levels. These results indicate the importance of the 8-oxoG repair system for cell viability.

## Author Summary

The parasite *Trypanosoma cruzi* is the causative agent of Chagas disease, a malady endemic throughout Latin America. Studying the DNA repair machinery of this parasite could provide us with good insights about *T. cruzi* biology and virulence. We focused on the 8-oxoguanine (8-oxoG) DNA lesion and its repair system. This lesion is considered particularly deleterious because it can generate DNA double strand breaks if inserted during the DNA replication. Our approach to investigating the importance of the 8-oxoG repair system in *T. cruzi* was to generate a parasite population expressing the *Escherichia coli* MutT enzyme, which is responsible for removing 8-oxo-dGTP from the nucleotide pool. Different parameters such as growth curves, cell infection experiments, antioxidants, enzymes expression, and DNA lesion quantification were used to study this modified parasite in comparison with a control WT population. We also characterized a gene in *T. cruzi* that has functional homology with the *E. coli* MutT gene. The overexpression of this gene in *T. cruzi* caused the same phenotypes observed when we expressed the heterologous gene. Overall, the results indicate the importance of this DNA repair enzyme for *T. cruzi* resistance to oxidative stress and improving its proliferative ability in the vertebrate host.

## Introduction

Oxidative stress is often defined as a situation in which the balance between oxidants and antioxidants is disrupted. The main source of oxidative stress in living organisms is reactive oxygen species (ROS), which are molecules, such as hydrogen peroxide, superoxide and hydroxyl radicals, that are derived from oxygen and are highly reactive toward biomolecules [1].

One of the most deleterious consequences of oxidative stress may be the formation of DNA lesions. Over 100 different types of oxidative DNA modifications have already been identified in the mammalian genome. However, due to its low redox potential, guanine (G) is the most vulnerable base [2]. The main product of G oxidation is 7,8-dihydro-8-oxoguanine (8-oxoG). Therefore, this product is the most common and best-characterized lesion created by ROS [3]. The strong relation between ROS production and 8-oxoG formation makes it a good and commonly used cellular biomarker of oxidative stress [4]. Its importance can be attributed to the fact that when 8-oxoG assumes its *syn* conformation, it is particularly mutagenic because of its strong ability to functionally mimic thymine. When 8-oxoG is inserted during DNA replication, it can generate double-strand breaks, which makes this lesion very deleterious [5].

The so-called GO-system is a three-component 8-oxoG repair pathway. In bacteria, MutT, MutY and MutM (also called Fpg) constitute this system [6]. The corresponding enzymes for humans are MTH1, MUTYH and OGG1, respectively [7]. MutT (or MTH1) hydrolyses 8-oxo-dGTP in the nucleotide pool, returning it to the monophosphate form so that it cannot be incorporated into DNA by polymerases [8], [9]. The enzymes MutM (or OGG1) and MutY (MUTYH) are responsible for repairing 8-oxoG paired with cytosine in the DNA or removing the adenine in the 8-oxoG:A mispair [2], [3], [10].

The focus of our group is the *Trypanosoma cruzi* parasite and how oxidative stress and DNA repair mechanisms could affect its cell viability. This flagellate parasite is responsible for the development of a malady called Chagas disease [11], a major public health problem in Latin America that affects over 10 million people, according to the World Health Organization. Currently, the disease is a world health concern due to globalization and population migration from endemic to non-endemic areas [12].

During its life cycle, *T. cruzi* infects two different hosts: a vertebrate mammalian host and an invertebrate insect vector from the Reduviidae family. This complex life cycle involves various different environments that the protozoan parasite has to address. The oxidative stress encountered in all these environments is one of the main threats to the trypanosome cell viability [12]–[16]. In the vertebrate host, cell infection depends initially on recruitment and fusion of lysosomes, which contribute to the formation of a stable parasitophorous vacuole [17]. Lysosomes are very acidic organelles with a high oxidative potential. Later, the parasite escapes from its vacuole into the cytosol, which may also represent a source of oxidative stress via the generation of ROS due to electron leakage from mitochondrial respiratory complexes [15], [18]. In the triatomine, the parasite develops inside the gut, where it is confronted with several changes, such as temperature, osmolality, nutrient supply, acidic or alkaline pH, as well as the oxidative stress caused by ROS production through hemoglobin degradation and nitrogen intermediate production by host defense mechanisms [13], [19], [20].

To cope with these oxidant environments, *T. cruzi* uses its defense machinery against oxidative damage. Its antioxidant machinery compose an efficient and well-compartmentalized network that acts in the detoxification of the reactive oxygen and nitrogen species produced during parasite-host cell interactions [14]. In addition, there is growing evidence that this antioxidant network may play an important role in parasite virulence [21]–[24].

Despite all of these antioxidant defenses, the parasite macromolecules, especially DNA, can undergo oxidative damage that can be deleterious if not repaired. The *T. cruzi* genome project identified several DNA repair pathway elements in the parasite genome [25]. Among these proteins, many of them act in response to oxidative lesions. Since the publication of the parasite's genome, some of this DNA repair enzymes have been characterized, as reviewed by Passos-Silva *et al.* [26]. However, some important elements of the DNA repair machinery, such as a MutT homolog, have yet to be identified. This prompted our group to transform a *T. cruzi* CL Brener clone with the *E. coli mutT* gene to investigate the importance of 8-oxoG during parasite infection of mammalian cells. MutT-expressing cells demonstrated more resistance to the oxidative stress caused by hydrogen peroxide (H_2_O_2_) treatment, as well as increased growth in *in vitro* and *in vivo* infection experiments. This difference could be due to a MutT enzyme product 8-oxo-dGMP, which can generate an oxidative stress signal, enabling the cells to overcome this stress. Furthermore, we demonstrated that *E. coli* MutT expression in *T. cruzi* reduces the amount of nuclear DNA lesions compared with control cells. In addition, we demonstrated that *T. cruzi* has a MutT homolog, termed here TcMTH, that is able to complement *mutT-*deficient bacteria and enhances parasite survival against oxidative treatment in the same manner that we observed for the bacteria gene.

## Materials and Methods

### Ethics statement

This study was conducted in strict accordance with the recommendations in the Guide for the Care and Use of Laboratory Animals of the Brazilian National Council of Animal Experimentation (http://www.cobea.org.br/) and the Federal Law 11.794 (October 8, 2008). All animals were handled in strict accordance with good animal practice as defined by the Internal Ethics Committee in Animal Experimentation of the Centro de Pesquisas René Rachou/Fundação Oswaldo Cruz (CPqRR/FIOCRUZ), Belo Horizonte (BH), Minas Gerais (MG), Brazil. The protocol number P-441-07 was approved by CEUA/FIOCRUZ with the license n° LW-61/12.

### Plasmids

The *pROCK_MutT_HYGRO* expression vector was generated by polymerase chain reaction (PCR) amplification of the *mutT* gene from AB1157 *E. coli* (GeneID: 5590913) and employed the following primers: 5′-TCTAGAATGAAAAAGCTGCAAATTGC-3′ (forward) and 5′-CTCGAGCTACAGACGCTTAAGCTTCGCA-3′ (reverse). The *pROCK_TcMTH_HYGRO* expression vector was generated by PCR amplification of the *tcmth* gene from *T. cruzi* strain CL Brener genomic DNA (GenBank: KC630985) and employed the following primers: 5′-TCTAGAATGGCCGCGATGACTGCGAC-3′ (forward) and 5′-CTCGAGTCAGCTGGAGTTTTCCTTGT-3′ (reverse). The PCR products were cloned into the pGEM-T Easy (Promega, Brazil) cloning vector. The *mutT* or *tcMTH* gene fragment was digested using *Xba*I and *Xho*I and then inserted into the *pROCK_HYGRO* vector, previously digested with the same restriction enzymes, to produce *pROCK_MutT_HYGRO* or *pROCK_TcMTH_HYGRO* [27].

### Parasite growth and transfection

*T. cruzi* CL Brener strain epimastigote forms were grown in liver infusion tryptose (LIT) medium (pH 7.3) supplemented with 10% fetal bovine serum (FBS, GibcoBRL, Invitrogen, CA, USA), streptomycin sulfate (0.2 g l^−1^) and penicillin (200000 units l^−1^) at 28°C. The parasite transfection was performed using electroporation following a previously described protocol [27]. The transfected parasites were cultured for 6 weeks in the presence of hygromycin (200 mg/mL, Sigma, MO, USA) for selection of parasites containing stably incorporated *pROCK_MutT_HYGRO* or *pROCK_TcMTH_HYGRO*.

### RNA purification and RT-PCR

*T. cruzi* total RNA purification was performed from 10^7^ epimastigotes using TRIzol (Invitrogen, Life technologies, CA, USA) reagent and treated with DNAse (Invitrogen) for DNA contaminant removal according to the manufacturer's instructions. The purified RNA was then used in a cDNA synthesis reaction with 500 ng oligo(dT)_12–18_, using the SuperScript III First-strand Synthesis System for RT-PCR (Invitrogen). The subsequent *mutT* fragment specific amplification was performed using the following primers: 5′-GTAGGTATTATTCGCAACGAGA-3′ (forward) and 5′-TTTCACCCATTTCAATTTTACCG-3′ (reverse). The negative control was processed in the same conditions as the other samples but without reverse transcriptase enzyme.

### Epimastigote growth and survival curves

Wild-type and transfected parasite growth curves were started at 5×10^6^ cells/mL. The cells were counted for 6 days. To test the resistance to H_2_O_2_, parasite cultures containing 1×10^7^ cells/mL were treated with 0, 150, 200 or 250 µM H_2_O_2_. The cells were counted after 72 h. The results are expressed as the percentage of growth compared with untreated cultures. In both experiments, the cell numbers were determined in a cytometry chamber using the erythrosine vital stain to differentiate living and dead cells. The experiments were performed in triplicate.

### QPCR analysis of DNA lesions

To compare the amount of DNA lesions from parasites transfected either with *pROCK* empty vector or *pROCK_MutT*, a quantitative polymerase chain reaction (QPCR) protocol adapted from Santos et al. [28] was employed. Epimastigotes cultures were grown on LIT medium under normal conditions. Parasites were harvested at 1×10^7^ cells/mL concentration by centrifugation at 3000 *g* for 10 min. Following that, high-molecular weight DNA extraction, quantification, QPCR amplification and result analyses were conducted as previously described [28]. This QPCR assay was performed by comparing the amplification of the DNA from the cells carrying the pROCK empty vector with the amplification of the cells expressing the MutT enzyme. Specific primers were used to amplify large and small fragments of the nuclear and mitochondrial DNA. The large nuclear fragment was amplified using the forward primer QPCRNuc2F (5′-GCACACGGCTGCGAGTGACCATTCAACTTT-3′) and the reverse primer QPCRNuc2R (5′-CCTCGCACATTTCTACCTTGTCCTTCAATGCCTGC-3′). The small nuclear fragment was amplified employing the internal primer QPCRNuc2Int (5′-TCGAGCAAGCTGACACTCGATGCAACCAAAG-3′) and the reverse primer QPCRNuc2R. The large mitochondrial fragment was amplified using the forward primer QPCRMitF (5′-TTTTATTTGGGGGAGAACGGAGCG-3′) and the reverse primer QPCRMitR (5′-TTGAAACTGCTTTCCCCAAACGCC-3′). The small mitochondrial fragment was amplified with the internal primer QPCRMitInt (5′-CGCTCTGCCCCCATAAAAAACCTT-3′). The small fragment (250 bp) amplification was used to normalize the amplification results obtained with the large fragments (10 kb), as the probability of introducing a lesion in a short DNA segment is very low, and this strategy eliminates the bias of changes in the proportion between nuclear and mitochondrial genomes. The normalized amplification of pROCK cells samples was then compared with MutT, and the relative amplification was calculated. These values were then used to estimate the average number of lesions per 10 kb of the genome, using a Poisson distribution. The final results are the mean of two sets of PCR for each target gene of at least 2 biological experiments.

### Trypomastigote differentiation and maintenance

For the differentiation of *T. cruzi* epimastigotes parasites to metacyclic trypomastigotes, an *in vitro* metacyclogenesis protocol was employed, using the chemically defined TAU and TAU3AAG media as previously described [29]. Following differentiation, the parasites were centrifuged, resuspended in DMEM (Gibco) supplemented with 2% FBS, 1% penicillin-streptomycin and 2 mM glutamine and used to infect LLC-MK2 monolayers. Once the infection was successfully established, the parasites were maintained in LLC-MK2 monolayers and purified as described previously [30].

### *In vitro* infection experiments in non-professional phagocytic cells

All *in vitro* infection experiments in non-professional phagocytic cells were performed using a mouse fibroblast cell lineage (WTCl3) derived from mouse embryonic fibroblasts [31]. Prior to infection, the cells were plated at 2.5×10^4^ cells/mL in medium containing 10% FBS on 24-well tissue culture plates containing 12-mm round coverslips and grown for 24 h at 37°C in a humidified atmosphere containing 5% CO_2_. Infection of WTCl3 fibroblasts with purified tissue culture trypomastigotes (TCTs) was performed for 30 min at 37°C at a multiplicity of infection (MOI) of 50 or 100 (for vacuole escape experiments). For hydrogen peroxide pre-treatment experiments, the parasites were treated with 50 µM H_2_O_2_ for 2 hours before incubation with cells. Immediately after cell infection, the cells were washed four times with phosphate-buffered saline (PBS) to remove extracellular parasites and reincubated with medium for 30 min (0 h time point) or different times according to the experiment, before fixation with 4% (wt/vol) paraformaldehyde/PBS overnight at 4°C.

### Immunofluorescence

After fixation, coverslips with attached cells were washed three times in PBS, incubated for 20 min with PBS containing 2% bovine serum albumin (PBS/BSA) and processed for an inside/outside immunofluorescence invasion assay as described previously [32]. Briefly, to distinguish extracellular parasites from intracellular ones, the cells were incubated for 50 min with a 1∶500 dilution of rabbit anti-*T. cruzi* polyclonal antibody in PBS/BSA, followed by 40 min incubation with Alexa-Fluor 546 goat anti-rabbit IgG (Invitrogen) diluted 1∶250 in PBS/BSA. For the vacuole escape kinetics experiments, after extracellular-parasite staining, the cells were permeabilized with PBS/BSA containing 0.5% saponin (PBS/BSA/saponin) for 20 min. Parasites associated with parasitophorous vacuole were labeled using a 50-min incubation with rat anti-LAMP1 antibody diluted 1∶50 in PBS/BSA/saponin, followed by 40-min incubation with a 1∶250 dilution of Alexa-Fluor 488 goat anti-rat IgG (Invitrogen) in PBS/BSA/Saponin. The cell and parasite DNA was stained with DAPI diluted 1∶1000. Slides were mounted and then examined on a Zeiss Axioplan-2 microscope. At least 300 cells were analyzed per coverslip in triplicate.

### Preparation of antibodies, protein extracts and western blotting analysis

The polyclonal antibodies anti-TcCPx and anti-TcMPx were obtained as previously described [33], [34]. Parasites (1.0–2.0×10^7^ cells/mL) were incubated or not (control) with H_2_O_2_ (50 µM) for 30 minutes in LIT medium. The cells were harvested by centrifugation (2700 g, 10 min) and resuspended in 80 µL of PBS/1 mM MgCl_2_, and an equal volume of lysis buffer (50 mM Tris-HCl pH 7.4, 1% Tween 20, 150 mM NaCl, 1 mM EGTA, 1 mM Na_3_VO_4_, 1 mM NaF, 0.1 mM PMSF, aprotinin 1 µg/mL; leupeptin 1 µg/mL) was added. The suspension was sonicated (Bandelin Sonoplus Homogenisatoren) for 10 cycles of 1 sec, with an interval of 1 sec and 30% max amperage. The material was kept for 2 h on ice and subsequently centrifuged (13,000 g, 4°C, 15 min). An equal volume of loading buffer was added to the protein extract (100 mM Tris-HCl, pH 6.8, 4% SDS, 0.02% bromophenol blue, 20% glycerol, 200 mM β-mercaptoethanol), and the samples were heated at 96°C for 4 min [35]. Protein concentration was determined by the Lowry technique in samples without loading buffer [36]. The protein extracts (30 µg) were separated and electroblotted onto a nitrocellulose membrane using the Trans-Blot SD Semi-Dry Electrophoretic Transfer Cell (Bio-Rad, CA, USA). The membranes were blocked by incubation with 5% instant nonfat dried milk in PBS 0.05% Tween 20 (PBS-T) for 1 h, washed and incubated in the presence of polyclonal antibodies raised against *T. cruzi* TcMPx (1∶2000) and TcCPx (1∶2500) for 2 h. After three 15 min washes with PBS-T, the membranes were incubated with HRP-linked anti-rabbit IgG (Cell Signaling Technology, MA, USA, 1∶5000 dilution) for 1 h at room temperature and washed three times with PBS [22], [37]. Bands were revealed using the Super Signal Detection Kit (Thermo Scientific, Pierce, IL, USA). Data were analyzed using the Scion Imaging Program and normalized using a loading control (anti-tubulin).

### *In vivo* infection experiments

Three-week-old female Swiss mice bred and maintained in the animal breeding units at CPqRR/FIOCRUZ were used. TCTs were purified, counted and diluted in DMEM to inoculate each animal with 5000 parasites equally via the intraperitoneal route. The experiments consisted of two groups of 6 animals for the WT and MutT parasites. Parasitemia was assessed by counting the trypomastigotes in 5 µl of tail vein blood of the infected mice, on alternate days from the 3^rd^ day p.i. until the time at which the parasites became undetectable. The number of parasites per mL was calculated as previously described [38].

### DNA sequencing and analysis

DNA sequencing reactions were performed using the ABI3130 automated capillary DNA sequencer (Applied Biosystems, Life Technologies, CA, USA) and BigDye Terminator cycle sequencing kit v3.1 (Invitrogen) by Myleus Biotechnology (www.myleus.com, MG, Brazil). The DNA Baser Sequence Assembler v3.2.5 (Heracle Biosoft) software was employed for contig assembly. The TcMTH sequences were deposited in GenBank and can be viewed under the accession number KC630985.The GenBank accession numbers of the other MutT homologs sequences used in this work are as follows: *Trypanosoma brucei* (*TbMTH*), accession number XP_822715.1; *Escherichia coli* (*EcMutT*), accession number ZP_12082602.1; and *Homo sapiens* (*hMTH1*), accession number BAA04013.1. Sequence alignments were performed using the Multalin [39] and Boxshade v3.21 (www.ch.embnet.org) interfaces. Motif and catalytic site identification were performed using the Conserved Domain Database of the NCBI [40].

### Mutation frequency analysis

The spontaneous mutation rate was determined using a rifampicin (rif) antibiotic resistance assay following a previously described protocol [41]. An *E. coli* BH600 (*mutT-*) bacteria strain was used for the heterologous complementation test, and the rif resistant mutants (r*if^R^*) frequency was determined. BH600 bacteria were transformed with the following pMAL-c2G (NE BioLabs Inc., MA, USA) constructs: pMAL_*mutT*, pMAL_*TcMTH* and pMAL empty vector. After confirming the transformants, 10^5^ cells were plated on 2×YT medium containing ampicillin (100 µg/mL), tetracycline (12.5 µg/mL) and IPTG (0.1 mM) to confirm the number of viable cells. The resistance assay was performed by plating the same cultures in 2×YT medium as described above supplemented with rif (100 µg/mL). At least 14 clones were plated in duplicates for each condition. The plates were grown for 16 hours at 37°C, and the number of *rif^R^* revertant colonies was counted. The mutation rate was calculated according to Lea & Coulson [42].

### Macrophage infection experiments

The macrophages used in this study were obtained from the peritoneal cavity as previously described [43]. Briefly, the cells were isolated from the peritoneal cavity of mice 3 days after injection of 2 mL of 3% thioglycollate medium (Biobras S.A., MG, Brazil) into the peritoneal cavity. The cells were resuspended in DMEM (Gibco), supplemented with 10% FBS, 1% penicillin-streptomycin and 2 mM glutamine. The macrophages were counted in a Neubauer chamber prior to seeding 5×10^5^ cells into each well of a 24-well plate and incubated at 37°C, 5% CO_2_ for 1 hour. The TCTs were purified, counted and diluted in DMEM medium, and infection was performed for 2 hours at an MOI of 5. Immediately after macrophage infection, the cells were washed four times with PBS to remove extracellular parasites and either fixed or reincubated with medium for 48 and 72 hours before fixation with methanol. Coverslips with attached macrophages were stained with Giemsa, and a minimum of 300 macrophages per coverslip were analyzed. The results were expressed as an infection index (% infected macrophages × number of amastigotes/total number of macrophage). The experiments were performed in triplicate.

### Statistical analysis

The statistical analyses in this work were performed using the GraphPad Prism 5.0 program (GraphPad Software Inc., CA, USA). Data are presented as the mean ± standard deviation (SD), and all experiments were repeated at least three times. Results were analyzed for significant differences using ANOVA or Student's *t* test. Statistical tests used are described at each figure legend. The level of significance was set at P<0.05.

## Results

### *EcMutT*-expressing *T. cruzi* cell line

**Figure 1:**
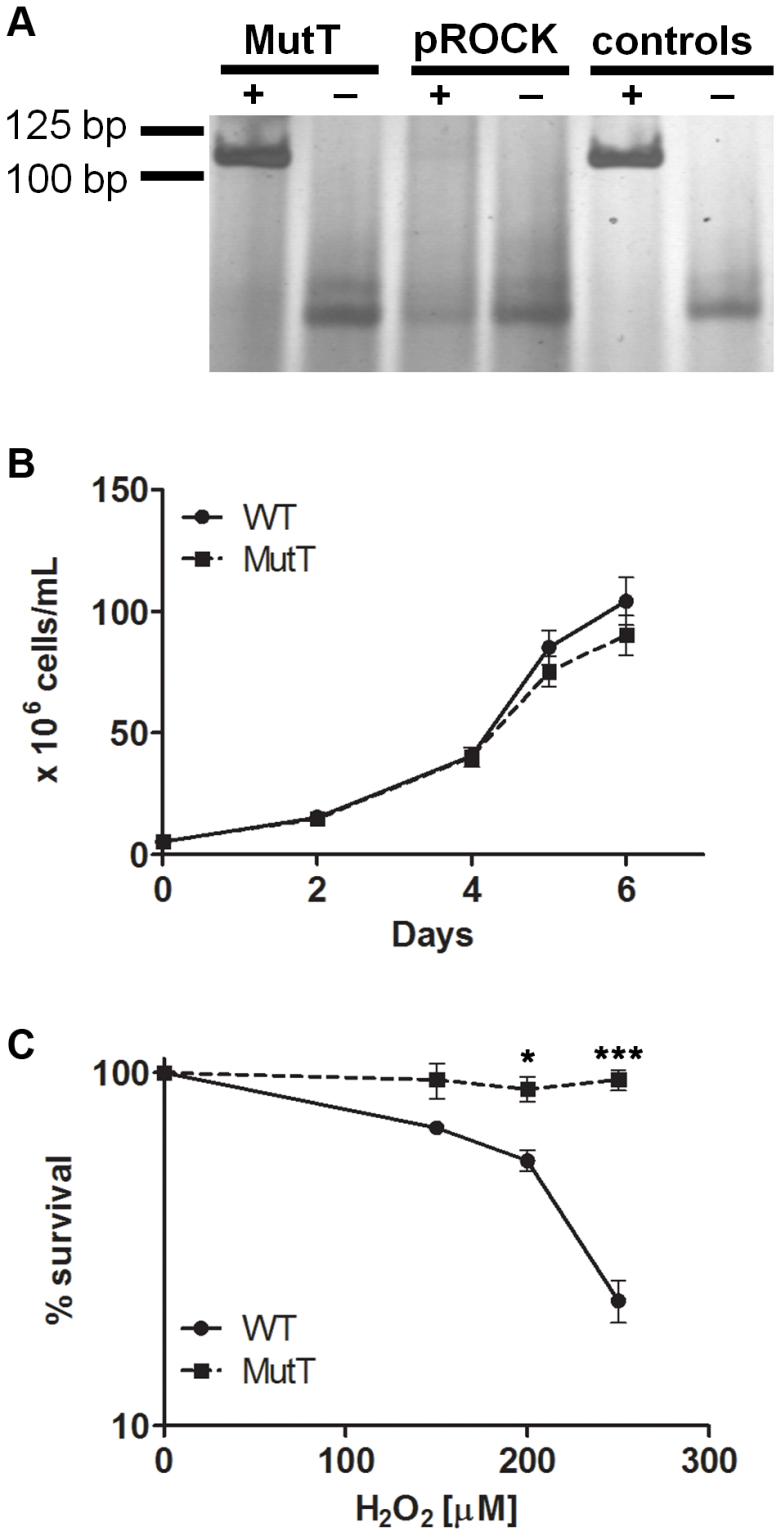
MutT heterologous expression did not alter *T. cruzi* growth but enhanced survival against H_2_O_2_. (A) Amplification of a 112-bp fragment of MutT from transfected parasite total RNA (**MutT +**) and positive controls (*E. coli* total DNA). **pROCK +**: total RNA from parasites transfected with the pROCK empty vector. **MutT -** and **pROCK -**: RT-PCR negative control without reverse transcriptase; **- control:** PCR control without template DNA. (B) WT and MutT parasites were grown in LIT medium and followed for 6 days until the stationary phase. Alternatively, WT and MutT were grown in LIT medium containing different concentrations of H_2_O_2_ for 3 days and then counted (C). Experiments were performed in triplicate. The survival percentage was measured in relation to untreated cells, and the bars represent the SD (*** P<0.001, * P<0.05, unpaired *t* test).

To investigate 8-oxoG lesion importance for the parasite, we generated a *T. cruzi* cell line stably expressing the MutT enzyme using the integrative vector *pROCK_HYGRO*. This vector integrates by homologous recombination at the β-tubulin locus, which contains several copies of the β-TUBULIN gene along with the α-TUBULIN gene [27]. The heterologous expression of *E. coli* MutT in the *T. cruzi* cell line transfected with the vector containing the *mutT* gene was confirmed through RT-PCR. The expected 112-bp fragment of the *mutT* gene was amplified from the cDNA of parasites transformed with the gene of interest and positive controls (Fig. 1A). To facilitate reading, the cell lines used in this work will be referred to as **WT** for wild-type CL Brener, **pROCK** for CL Brener transformed with the *pROCK* empty vector, and **MutT** for the parasites that express the *E. coli* MutT enzyme.

### MutT-expressing epimastigotes grow similar to the wild type but are more resistant to H_2_O_2_ treatment

After MutT heterologous expression was confirmed, we went on to investigate the behavior of epimastigotes growth *in vitro*. The expression of MutT did not alter the parasite growth curve in normal conditions (Figure 1B). To verify if MutT expression alters *T. cruzi* response to oxidative stress, the epimastigote cultures were treated with H_2_O_2_, and cell viability was determined after 3 days. As shown in Figure 1C, MutT parasites displayed greater resistance to H_2_O_2_ toxicity than WT cells (P<0.05). In the presence of 250 µM H_2_O_2_, approximately 95% of the transfected cells survived in contrast to only 22% of the wild-type cells.

### MutT-expressing cells contained fewer nuclear DNA lesions

**Figure 2:**
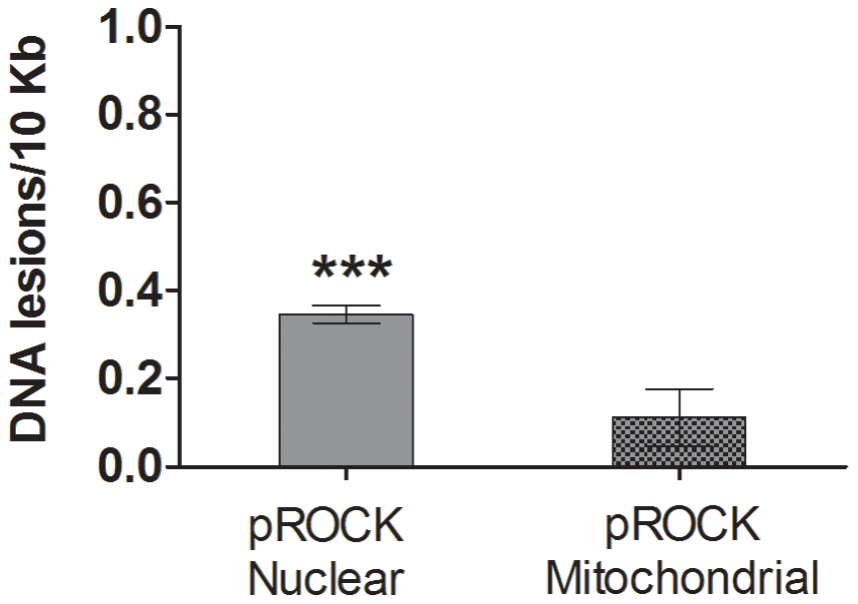
Analysis of pROCK parasite DNA lesions compared with MutT parasites through a QPCR assay. Specific primers were used to amplify large and small fragments of the nuclear and mitochondrial DNA of untreated cells. Normalized amplification fluorescence values are in Table 1. This values were used to estimate the relative amplification of pROCK cells compared to MutT. The pROCK average number of lesions per 10 kb of the genome in relation to MutT parasites was then estimated by the relative amplification calculated (*** P<0.0001, unpaired *t* test).

The QPCR technique was employed to compare the extent of DNA damage in the genome of the parasite populations used in this work. The normalized amplification of pROCK cells was compared to MutT (Table S1), and the relative amplification was calculated. These values were used to estimate the average number of lesions per 10 kb of the genome in relation to MutT parasites. As Figure 2 shows, pROCK cells presented 0.34 more DNA lesions per 10 kb in the nuclear genome compared with MutT cells (P<0.0001, unpaired *t* test). The difference in DNA lesions in the mitochondrial genome for the two parasite populations was not significant.

### The kinetics of invasion and escape from the parasitophorous vacuole are not altered by MutT expression

**Figure 3:**
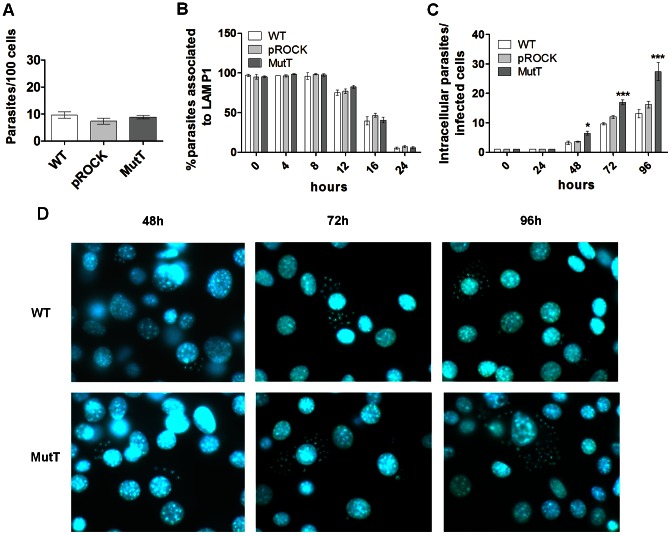
MutT expression enhances *in vitro* intracellular growth. Murine fibroblasts were exposed to trypomastigotes (MOI of 50) for 30 minutes. Monolayers were washed to remove extracellular parasites and either fixed (PFA 4%) or incubated with fresh medium without parasites for different times. Slides were stained by immunofluorescence and analyzed in fluorescence microscope. (A) Parasites infectivity was determined by counting the number of internalized trypomastigotes per 100 cells right after cell exposure to parasites. (B) Parasitophorous vacuole escape kinetics were determined by analyzing the number of parasites co-localizing with LAMP, a lysosomal marker, at different time points for 16 hours after invasion. (C) Number of intracellular parasites per infected cell for WT, pROCK and MutT infected cultures from 24 up to 96 hours post-infection (*** P<0.001, * P<0.05, two-way ANOVA test with Bonferroni post-test). (D) Representative images of murine fibroblasts infected either with WT or MutT at an MOI of 50, cells and parasites DNA were labeled with DAPI.

The invasion and intracellular development of WT, pROCK and MutT parasites in mammalian cells were assayed by the *in vitro* infection of murine fibroblasts. These experiments allowed us to determine if MutT heterologous expression influenced any step of parasite infection of mammalian cells. To investigate the influence of MutT on *T. cruzi* invasion processes in host cells, murine fibroblasts were exposed to parasites for 30 min, washed to eliminate extracellular parasites, and fixed after a 30 min incubation in fresh medium. An analysis of the number of internalized parasites per 100 counted cells indicated that there was no difference in the invasion rates for the three parasite populations tested, as confirmed by a one-way ANOVA test, indicating that MutT heterologous expression does not affect the invasion process (Figure 3A).

We also performed immunostaining of LAMP-1 (a lysosomal membrane protein found in the parasitophorous vacuole) in infected cultures to evaluate parasite vacuole escape kinetics over the first 24 hours following parasite invasion. During the first 8 hours of infection, 100% of internalized parasites were associated to LAMP-1, indicating they were still inside the parasitophorous vacuole (Figure 3B). In the subsequent hours, a decrease in the number of LAMP-1-associated parasites was observed, indicating parasite escape from its vacuole, which occurred at the same rate for the three parasite populations (Figure 3B). These results indicate that MutT heterologous expression also did not influence *T. cruzi* trypomastigote intracellular traffic.

### *In vitro* intracellular growth is enhanced by MutT expression

Following vacuole escape, *T. cruzi* differentiates into the amastigote replicative form and starts replication in the host cell cytoplasm. To investigate whether MutT heterologous expression would affect intracellular replication, fibroblast cultures infected with WT, pROCK or MutT parasites were followed for 96 hours post-infection. Intracellular development was determined by counting the number of intracellular parasites per infected cell at 24, 48, 72 and 96 hours post-infection. During the first 24 h of infection WT, pROCK and MutT cells behaved similarly with the same counts of intracellular parasites per infected cell. However, an increased number of intracellular parasites could be observed for MutT-infected cells at 72 h post-infection, compared with WT or pROCK parasites. This difference became even more evident at 96 h post-infection (Figures 3C and 3D). In addition, the number of trypomastigote forms released by MutT-infected cultures after 96 hours was higher than controls (data not shown). These data not only demonstrate that MutT parasites are able to complete the parasite intracellular life cycle but also demonstrate that the heterologous expression of *mutT* gene influences parasite intracellular development by increasing its replication rate.

**Figure 4:**
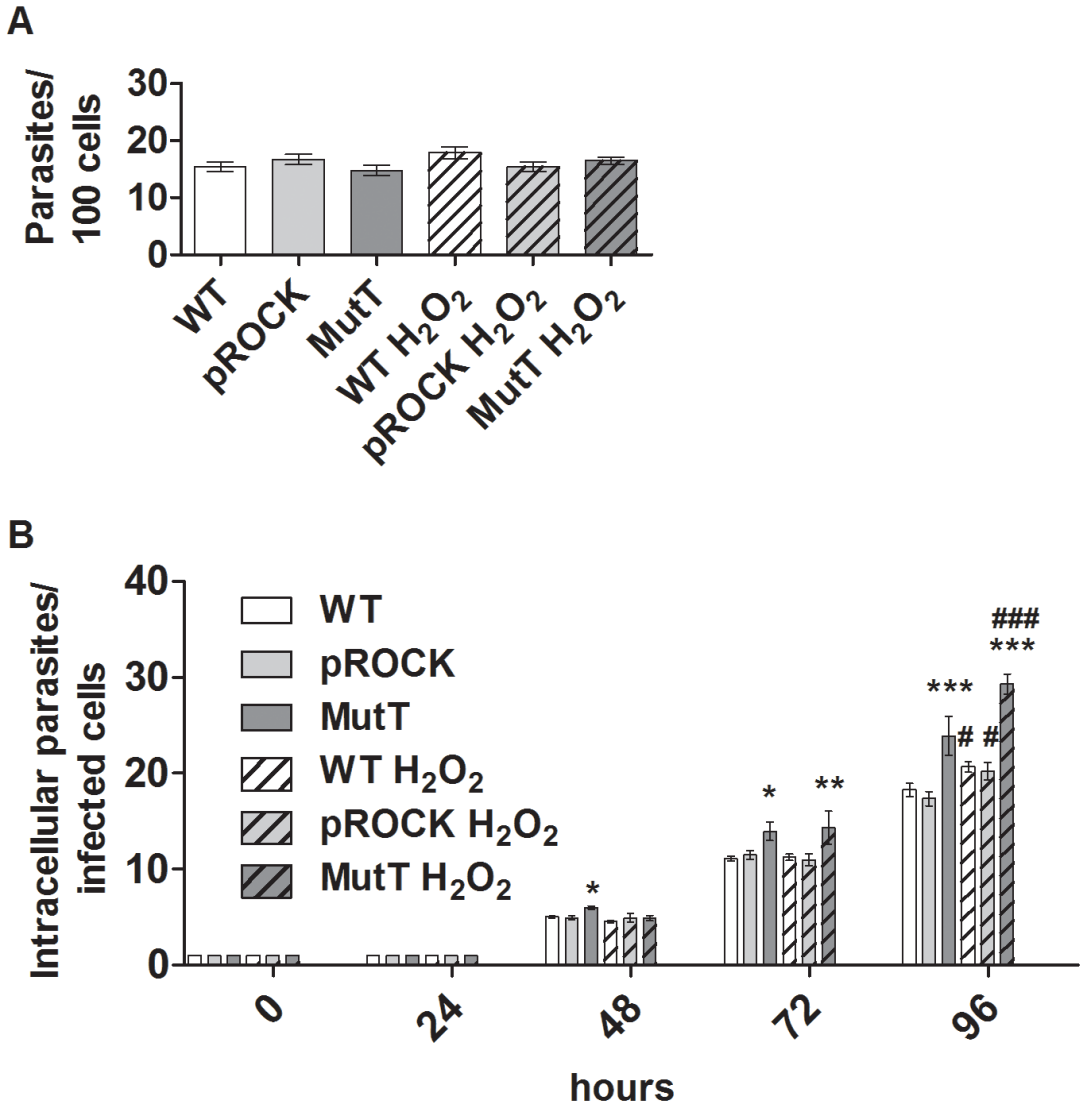
H_2_O_2_ treatment does not affect MutT parasite infectivity and replication. (A) Parasite infectivity after H_2_O_2_ treatment was determined by pre-treating parasites with 50 µM H_2_O_2_ before the invasion assay and counting the internalized trypomastigotes per 100 cells right after cell exposure to parasites. (B) Number of intracellular parasites related to time for cultures infected with WT, pROCK or MutT parasites pre-treated or not with 50 µM H_2_O_2_. The asterisk symbol (*) refers to significant differences of MutT parasites compared with controls, and the hash mark (#) indicates a significant difference of parasites in the H_2_O_2_-treated group and the untreated parasites (*** P<0.001, ** P<0.01, * P<0.05, ### P<0.001, # P<0.05, two-way ANOVA test with Bonferroni post-test).

To evaluate if the increased intracellular replication rate of MutT parasites could be attributed to oxidative stress resistance, parasites were incubated with 50 µM H_2_O_2_ prior to infection. Oxidative stress inflicted on the parasites by H_2_O_2_ did not change the parasite invasion rate compared with non-treated parasites and, as expected, was the same among the different parasite populations (Figure 4A).The cultures were followed during the 96 hours following parasite invasion. Intracellular growth curves of untreated parasites were very similar to the previous experiment in which the number of intracellular parasites per cell was higher for MutT-infected cultures 48 h post-infection (Figure 4B). Pre-treatment of MutT parasites, however, further increased the number of intracellular parasites per infected cells when compared with MutT non-treated parasites (Figure 4B). The intracellular replication rate kinetics of the WT and pROCK parasites were affected by previous treatment of parasites with hydrogen peroxide, and these cells were able to grow better after hydrogen peroxide treatment. These results might suggest that MutT cells present improved recovery from the oxidative damage inflicted to their DNA through hydrogen peroxide treatment, and pre-treatment prepared these cells to face the oxidative stress that occurs during cell infection.

### Antioxidant enzyme protein levels are influenced by MutT expression

**Figure 5:**
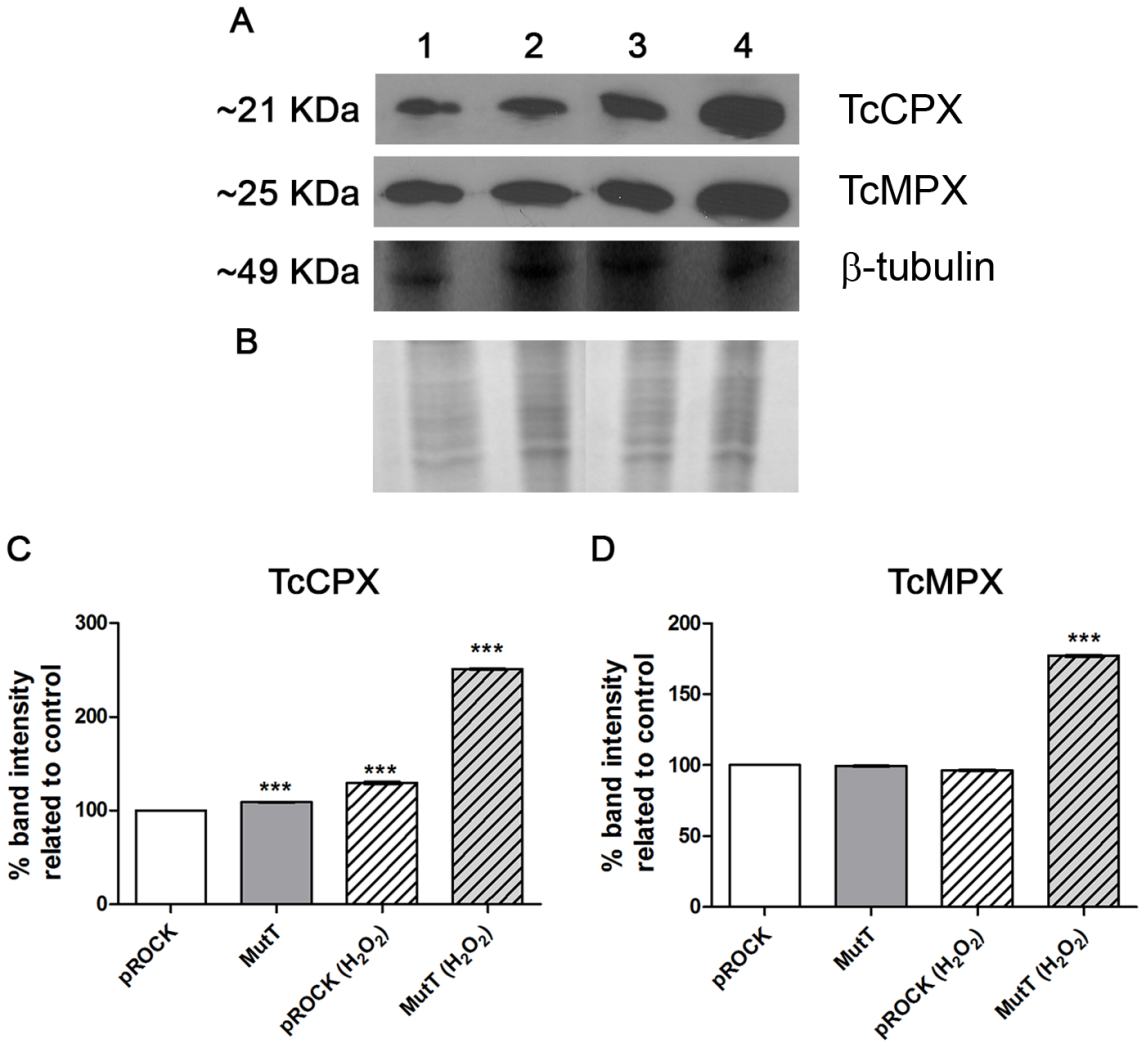
TcCPx and TcMPx expression in pROCK or MutT parasites. *T. cruzi* epimastigotes lysates were prepared with exponential phase cultures after 20 min incubation or not with 50 µM H_2_O_2_. Protein extracts were quantified by Bradford assay and resolved by SDS-PAGE (A) (30 µg protein/lane). Western blot analysis of TcCPx and TcMPx from non-treated pROCK (1) and MutT (2), or 50 µM H_2_O_2_-treated pROCK (3) and MutT (4) parasites. β-tubulin was used as loading control. (B) Coomassie blue staining of the protein extracts. The best representative of three independent experiments is shown. The signal intensity obtained for each studied enzyme from pROCK untreated control was set to 100%, and the enzyme levels from the others parasites were evaluated as a percentage of the control. The results were expressed in graphs for each enzyme: TcCPx (C) and TcMPx. (D) The asterisk symbol (*) refers to significant differences from the pROCK non-treated control (*** P<0.001, one-way ANOVA test with Bonferroni post-test).

As pROCK and MutT parasite populations markedly diverge in their resistance to oxidative stress and growth, we decided to analyze their cytosolic and mitochondrial tryparedoxin peroxidases (TcCPX and TcMPX) protein levels through western blotting experiments (Fig. 5A). Band densitometry revealed that MutT parasites presented higher levels of TcCPX (Fig. 5C) compared with the pROCK control (P<0.001). After submitting these parasites to a non-lethal dose of H_2_O_2_ (50 µM), pROCK cell TcCPX protein levels increased 29%, and MutT treated parasites presented a 2.5-fold increase compared with pROCK untreated controls (P<0.001). The TcMPX expression profile was slightly different, as neither untreated MutT nor treated pROCK parasites differed from the untreated pROCK control. However, MutT parasites treated with 50 µM H_2_O_2_ presented an approximately 70% increase in TcMPX expression level compared with pROCK controls (P<0.001).

### MutT expression improves parasites growth *in vivo*

**Figure 6:**
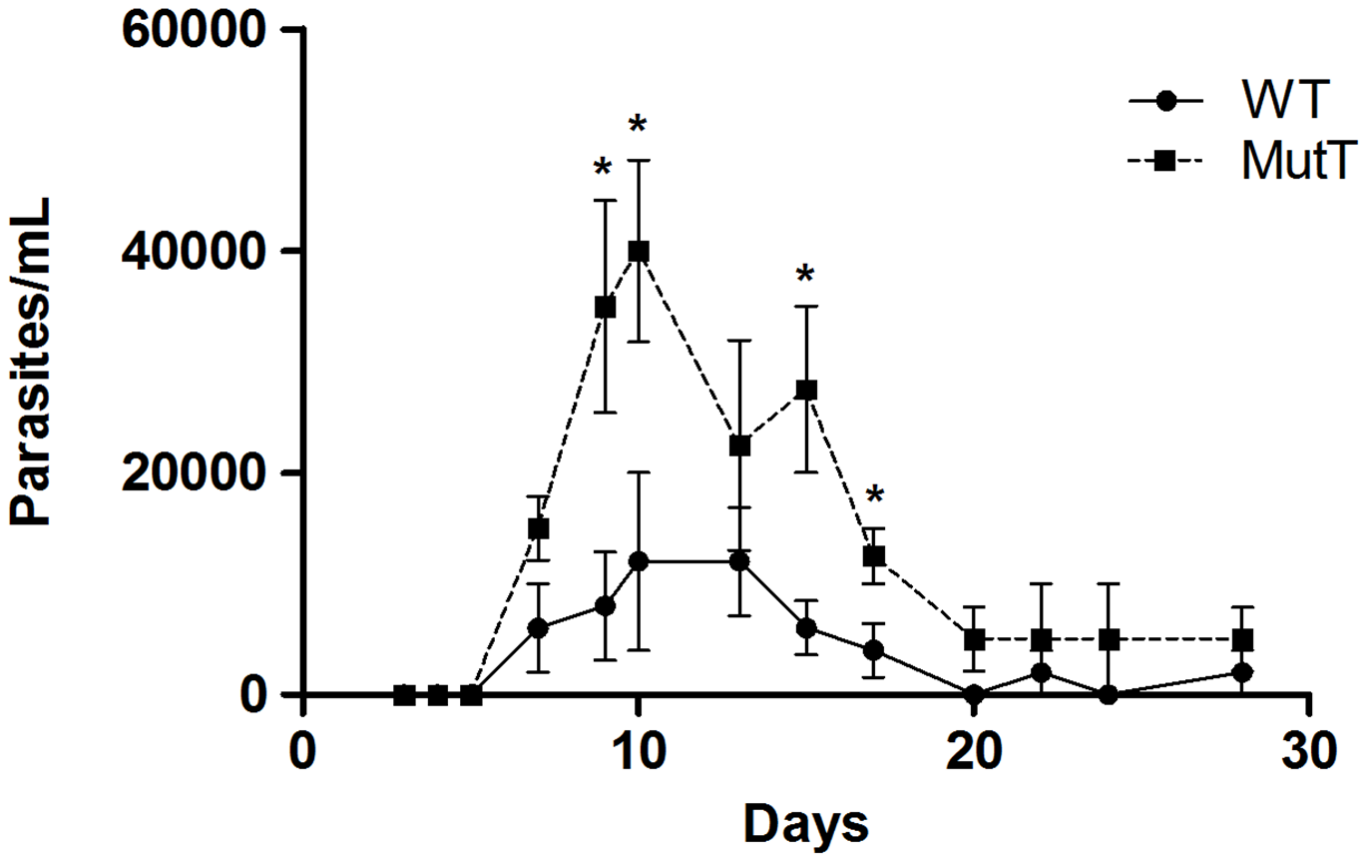
Parasitemia from animals infected with MutT or WT parasites. Three-week-old female Swiss mice were infected via intraperitoneal route with 5000 TCTs. The parasitemia was evaluated for up to 28 days post-infection by counting bloodstream form of parasites in tail vein blood. Parasitemia levels are expressed as the arithmetic mean of five mice per group (representative of three independent experiments). Significant differences between the two curves are represented in the graph (* P<0.05, unpaired *t* test).

To investigate whether the increase in the intracellular growth rate observed for the MutT parasites would also affect development *in vivo*, Swiss mice were infected with 5000 TCTs of WT or MutT parasites, and parasitemia was evaluated from day 3 post-infection (p.i.). The data obtained revealed that MutT-infected mice presented significantly higher parasitemia (P<0.05) compared with animals infected with WT parasites (Figure 6). This difference was more prominent at 9 days p.i., and animals infected with MutT parasites sustained higher parasitemia levels over the time course of infection compared with WT-infected mice.

### *T. cruzi* has a putative MutT homolog, TcMTH

After a detailed search of the *T. cruzi* genome database using the Nudix motif as query, a highly conserved 23-residue sequence found in the Nudix hydrolase superfamily [44], we identified a putative MutT homolog. This gene was successfully amplified from *T. cruzi* CL Brener strain genomic DNA, and the gene sequence was deposited in GenBank (accession number KC630985). Figure S1 shows the alignment of the *TcMTH-*deduced amino acid sequence and orthologs from other organisms. Sequence analysis revealed that TcMTH is a 306-amino acid protein that possess a perfect Nudix motif, a catalytic site and a divalent cation interaction residue, typical of an 8-oxo-dGTP pyrophosphohydrolase MutT enzyme.

### TcMTH complements *mutT-* bacteria

To investigate the activity of TcMTH *in vivo*, we examined its ability to complement the hypermutator phenotype of a *mutT^−^* bacterium strain (BH600). The *TcMTH* gene was cloned into an IPTG-inducible expression vector (pMAL) as well as the bacterium *mutT* gene. The *mutT^−^* strain was transformed with the constructs *pMAL_TcMTH*, *pMAL_MutT* and the *pMAL* empty vector. As seen in Table 1, the mutation rate was higher in *mutT-*deficient cells transformed with pMAL empty vector when compared with BH600 cells transformed with either EcMutT or TcMTH genes. Reverting the *mutT* deletion by transforming cells with *pMAL_MutT* vector resulted in a 6-fold decrease in mutation rate, whereas cells expressing the *T. cruzi* MutT homolog presented a 7-fold decreased mutation rate.

**Table 1:** Heterologous complementation assay with BH600 (*mutT-*) bacteria.

	Median	Mean +/− SD	Mutation Rate	Decrease variation of mutation rate
pMAL	99.5	135.92+/−120.17	7.85×10^−8^	0
pMAL_MutT	8.25	20.04+/−27.08	2.23×10^−8^	6×
pMAL_TcMTH	5.25	17.79+/−24.35	8.64×10^−9^	7×

*mutT*-deficient bacteria were transformed with three different constructs: pMAL empty vector, pMAL_MutT and pMAL_TcMTH. Transformed bacteria were grown in plates containing ampicilin, tetracycline and IPTG to confirm the number of viable cells. The mutation rate was determined by counting the number of rifampicin resistant revertant colonies after plating bacteria in medium containing rifampicin. The table summarizes the results for the spontaneous mutation of 14 clones in duplicates for each construction.

### Parasites overexpressing TcMTH are more resistant to H_2_O_2_

**Figure 7:**
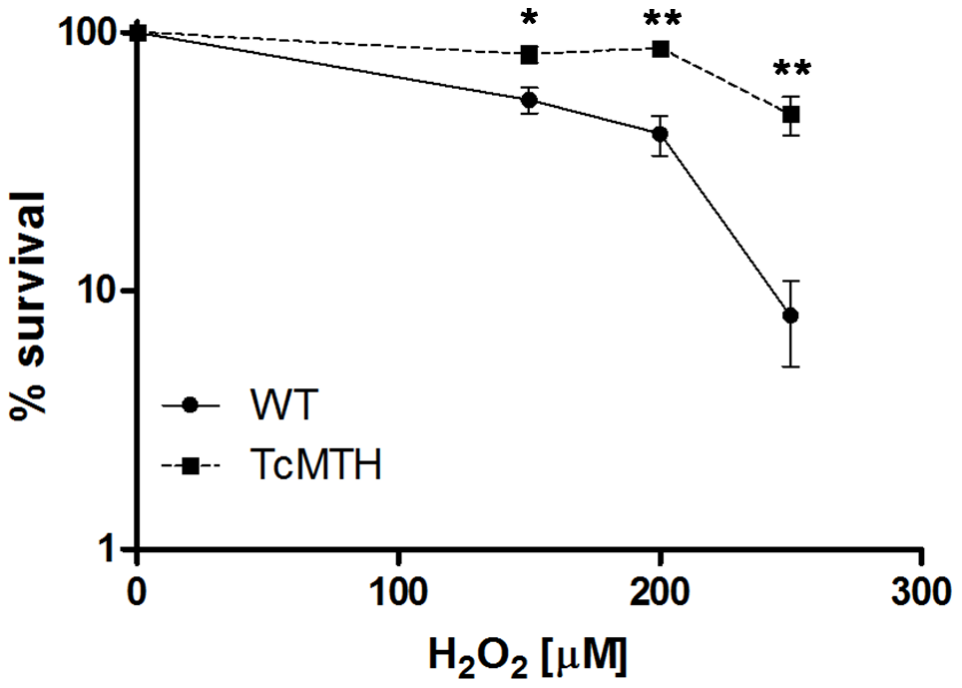
*T. cruzi* MTH overexpressors survival after H_2_O_2_ treatment. _2_O_2_ doses, and after 3 days, the parasites were counted. Survival percentage was measured in relation to untreated cells (** P<0.01, * P<0.05, unpaired *t* test).

We generated a *T. cruzi* population overexpressing TcMTH using the integrative vector pROCK carrying the gene *TcMTH* and analyzed their response to hydrogen peroxide treatment. As shown in Figure 7, TcMTH overexpression enhanced survival to H_2_O_2_ treatment, when compared to WT cells. Thus, MutT heterologous expression and TcMTH overexpression produced the same effects in *T. cruzi* epimastigotes, namely improving the parasite response to oxidative stress.

### Parasites with enhanced 8-oxod-GTP pyrophosphohydrolase activity presented improved growth in macrophage cultures

**Figure 8:**
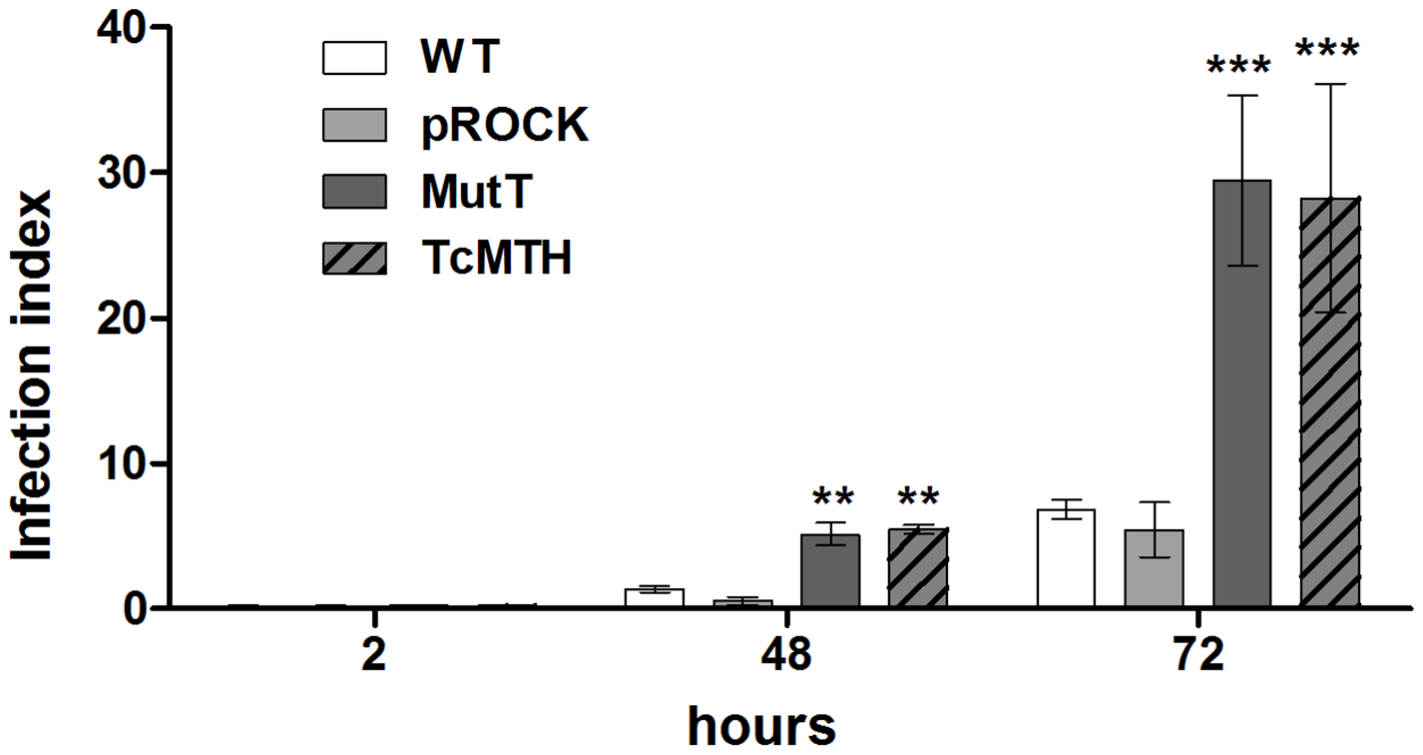
Macrophage infection experiment. Inflammatory macrophages obtained from peritoneal cavity 3 days after injection of thioglycolate were subjected to infection with WT, pROCK, MutT or TcMTH TCTs (MOI 5). The cells were washed to remove extracellular parasites and either fixed or reincubated with medium for 48 and 72 hours. The slides were stained with Giemsa and counted to determine the infection index (percentage of infected macrophages multiplied by the average number of amastigotes per macrophage) for each parasite population (*** P<0.001, ** P<0.01, one-way ANOVA with Bonferroni post-test).

Finally, we compared MutT-expressing parasites and TcMTH-overexpressing parasites with WT and pROCK control parasites in macrophage infection experiments. The results indicated that the modified parasites (MutT and TcMTH) presented enhanced replication inside murine inflammatory macrophages when compared with control parasites (Figure 8). The infection index obtained demonstrated that MutT parasites display the same advantage in growth exhibited in the fibroblast and mouse infection experiments, and TcMTH cells behave similarly to MutT-expressing parasites.

## Discussion

In the present work, we addressed the importance of 8-oxo-dGTPase activity in *T. cruzi*, which was previously supposed to lack a MutT homolog in its genome. Considering the different oxidative stress environments *T. cruzi* has to address through its entire life cycle [14], [45], [46], one would expect that oxidized nucleotides might be generated in this organism. These oxidative stress conditions may cause serious damage to the parasite DNA that would represent a threat to *T. cruzi* cell viability if not properly repaired.

In this study, we created a *T. cruzi* parasite population expressing *E. coli* MutT mRNA (MutT). The MutT parasite population exhibited similar behavior to the wild-type (WT) parasites in terms of the epimastigote growth curve in LIT medium. This was not totally unexpected because, unlike the invertebrate host niche, the culture medium will not contain oxidative stress sources, such as free radicals from heme production, usually found in the triatomine gut. Therefore, in this condition, the parasite intrinsic DNA oxidative damage repair system might be sufficient to allow satisfactory growth. However, when we performed a hydrogen peroxide survival curve in LIT medium, the MutT population was more resistant to the oxidant treatment than WT parasites. In addition, the QPCR analysis of DNA lesions demonstrated that parasites expressing the bacteria MutT presented fewer lesions in the nuclear DNA compared with the pROCK controls. These results indicate that the expression of exogenous MutT allows an improved control of oxidized nucleotide incorporation to DNA, preventing lesions that can arise from it, and these results emphasize the importance of 8-oxo-dGTP hydrolysis. These results are in agreement with previous data that showed the importance of oxidized nucleotide clearance in bacteria [47]. It was previously shown that major classes of bactericidal antibiotics act using a common pathway that produces hydroxyl radicals and that after antibiotic stress, *E. coli* cells maintain a constant level of MutT [48]. Foti *et al.* [47] demonstrated that much of the cell death caused by bactericidal antibiotics is related to oxidation of guanine to 8-oxo-guanine in the nucleotide pool. Their findings suggest that nucleotide sanitizing enzyme up-regulation may improve cell fitness by decreasing double-strand breaks and thus lethal lesions. Another possibility is that the MutT-expressing parasites produce more 8-oxo-dGMP that could serve as a signal for oxidative stress, making the cells modify their metabolism to respond to this stress.

Infectivity in murine fibroblasts of MutT-expressing parasites in comparison with control (WT and pROCK) populations was also tested. Our results indicate that during the first 24 hours of infection, while the parasites are still in the trypomastigote form, the three parasite populations tested presented similar invasion and parasitophorous vacuole escape behavior. These results can be explained by the fact that during this period, the parasites are in a non-replicative form, and the target for MutT, 8-oxo-dGTP, would not be incorporated in DNA until the next round of replication. Thus, the effect of MutT heterologous expression in *T. cruzi* did produce an apparent phenotype in the trypomastigote form. On the other hand, after parasites differentiated into the amastigote replicative form, MutT parasites exhibited faster replication rate than controls. This difference was evident after 48 hours of infection, when we could observe parasite intracellular multiplication. This result corroborates the findings of Gupta *et al.* [15] that showed an exponential increase in ROS production in *T. cruzi-*infected cells up to 48 hours post-infection. Thus, MutT-expressing parasites could be growing faster because they are better able to combat the oxidative stress present in the murine fibroblast cytoplasm. Moreover, treating parasites with hydrogen peroxide prior to infection augmented parasite fitness because at 96 hours p.i., the treated parasites presented increased growth compared with untreated parasites, and the MutT-treated parasites displayed an even higher growth rate increase.

To investigate these results, we analyzed *T. cruzi* antioxidant enzyme protein levels through western blotting experiments. The results indicate that *E. coli* MutT expression influenced peroxidases expression levels. The cytosolic peroxidase protein level increased for both pROCK and MutT parasites after H_2_O_2_ treatment. However, the MutT parasites presented a more pronounced increase, with a 2.5-fold change compared with the pROCK untreated controls. The mitochondrial peroxidase protein level displayed no variation in pROCK parasites after H_2_O_2_ treatment, but the MutT parasites presented a 70% increase after oxidative treatment. Overall, these data demonstrate that MutT parasites enhanced the antioxidant proteins levels after the oxidative treatment to much higher levels than pROCK parasites, indicating that MutT expression can influence antioxidant enzyme expression.

Our hypothesis for MutT differential behavior is that the formation of 8-oxo-dGMP by MutT serves as a signal and stimulates cells to become more proficient in responding to oxidative stress, changing antioxidant proteins expression and/or other BER and DNA repair pathways enzymes. The oxidative stress inflicted by H_2_O_2_ treatment would lead to the formation of excess 8-oxo-dGTP, which could be hydrolyzed by the heterologous MutT. The product of this reaction, 8-oxo-dGMP, or another secondary metabolite from this process, could be acting as a second messenger to the cell, indicating the presence of oxidative stress and shifting the parasite in a way that makes it more apt to act in response to that. The participation of the 8-oxoG repair pathway in cellular signaling pathways was recently demonstrated by Boldogh *et al.* [49]. Their study demonstrated that the 8-oxoG excised from DNA by OGG1 binds back to the enzyme at a nonsubstrate site with high affinity. The OGG1-8-oxoG complex formed interacts with the Ras GTPase enzyme, acting as a guanine nucleotide exchange factor. This interaction increases the Ras-GTP bound forms, enabling this GTPase to activate signaling pathways, including those that may modulate the expression of enzymes involved in oxidative stress response.

The results presented here suggest that pre-treatment may induce an adaptive response to the oxidative stress encountered in the host cell, preserving its genetic content integrity during the oxidative stress and allowing the parasite to replicate faster. Similar behavior has been documented previously for bacteria [50], yeast [51], mammalian cells [52], and *T. cruzi* [23]. The latter study reported that there is an adaptation to oxidative stress when parasites are treated with low non-toxic concentrations of H_2_O_2_ and then submitted to higher, though sub-lethal, concentrations of H_2_O_2_. Apparently, pre-treatment induces an increase in TcCPX levels from the parasite antioxidant network, preparing the parasite to address the fluctuating levels of ROS [23]. The recent findings of Nogueira *et al.* [53] confirm that low levels of ROS production induced by heme in *T. cruzi* epimastigotes favors parasite proliferation via a Ca^2+^ calmodulin kinase II (CaMKII)-like pathway. In addition, antioxidant activity (urate and GSH) inhibited heme-induced ROS and parasite proliferation [53]. In addition, Paiva *et al.* [54] demonstrated that maintenance of high parasite burden during *T. cruzi* infection might be dependent of oxidative stress generation.

The *in vivo* infection experiment results confirmed our *in vitro* findings, demonstrating that MutT parasites replicate faster in animal models, as demonstrated by the higher parasitemia found in MutT infected mice. The *in vitro* experiments had shown that MutT expression favors amastigote pronounced growth inside host cell, which reflected on the number of released trypomastigote forms (data not shown). *In vivo*, the enhanced intracellular growth could also promote substantial release of trypomastigotes in the bloodstream, which would explain the higher parasitemia levels.

Altogether, these results suggest that *E. coli* MutT heterologous expression in *T. cruzi* allows parasites to cope with oxidized nucleotides more efficiently in both the replicative amastigote and epimastigote forms. Nevertheless, these results do not deny the possibility of a *MutT-like* activity in *T. cruzi*. Indeed, previous studies have shown that despite the action of various enzymes and metabolic pathways in the control of oxidative stress effects, cellular defenses may be insufficient in a situation of intense stress, as in the hydrogen peroxide treatment [55]. However, these defenses could be amplified by the expression of one or more of its elements in higher levels. Thus, the enhanced efficiency of oxidative stress response presented by the recombinant parasites could be a consequence of high levels of MutT heterologous expression in either cells that were devoid of 8-oxo-dGTPase activity or cells that had low, yet sufficient, levels of this activity in the absence of oxidative stress.

Recently, our research group discovered a *T. cruzi* MutT homolog candidate (termed here as TcMTH) that was not annotated in the parasite genome project [25]. The DNA sequence and the predicted protein sequence for this candidate were characterized, and the elements for an enzyme from the Nudix superfamily were identified. This is a family of enzymes that displays great catalytic versatility [44], which could indicate that the candidate is not necessarily a MutT pyrophosphohydrolase. However, we demonstrated in this study that this possible TcMTH is able to complement a *mutT-*deficient bacteria strain, diminishing its mutation rate, which strongly suggests a case of functional homology. Moreover, *T. cruzi* parasites overexpressing this gene demonstrated increased resistance to oxidative stress, as previously shown for parasites heterologously expressing the *E. coli* MutT.

The recombinant parasite populations generated in this study were compared in a macrophage infection experiment. The infection index obtained for this experiment demonstrated that MutT as well as TcMTH parasites presented increased growth inside inflammatory macrophages when compared with the control parasites. This result might be additional evidence that TcMTH is indeed a MutT homolog in *T. cruzi*. Therefore, assuming that *T. cruzi* is endowed with an 8-oxo-dGTPase specific activity, we can speculate that its level might be low due to the wild-type sensitivity to oxidative stress treatment.

The persistence of oxidized nucleotides in the nucleotide pool as a consequence of this low activity could increase the 8-oxoG frequency in DNA. This could be compensated for by modulating the activity of other enzymes associated with the 8-oxoG repair pathway, such as TcOGG1, or alternative glycosylases. One example would be the Nei glycosylase that has been reported to act in oxidative lesions (including 8-oxoG) during transcription and/or replication [56]. The *T. cruzi* genome contains the sequence (Tc00.1047053506357.80) of a hypothetical NEIL glycosylase that has not been characterized and could be compensating for MutT low activity. Alternatively, *T. cruzi* translesion synthesis processes could neutralize the effects of 8-oxoG incorporation in DNA. The nuclear DNA polymerase TcPolη and the mitochondrial DNA polymerase TcPolκ are capable of bypassing 8-oxoG in an error-free manner, and parasites that overexpress TcPolη or TcPolκ demonstrate increased resistance to H_2_O_2_ treatment [57], [58]. In addition, transcription-coupled repair (TCR) [59] and mismatch repair (MMR) [60], [61] elements have been reported to participate in 8-oxoG repair.

Despite of all this repair machinery that could be involved in DNA oxidative damage repair, the results presented here indicate that the MutT 8-oxo-dGTPase activity can be crucial for guaranteeing parasite replication efficiency. In summary, we demonstrated that modifying the expression levels of an element from the 8-oxoG repair system can influence the parasite cell viability in both replicative phases, as previously observed for the parasite antioxidant machinery [62]. Considering that oxidative stress is important to *T. cruzi* proliferation in both replicative forms as demonstrated by Paiva *et al.* [54] and Nogueira *et al.* [53], mechanism for counteracting DNA oxidative damage would be of great importance for this parasite. *T. cruzi* DNA repair machinery presents some particular features from typical eukaryotic DNA repair machinery. Studying this particularities might indicate a path for drug development against Chagas disease .

## Supporting Information

Figure S1 **Alignment with the predicted** ***TcMTH*** **product and orthologs.** Amino acid sequence comparison of the predicted product of the *MTH* gene from *Trypanosoma cruzi* (TcMTH), *T. brucei* (TbMTH), *Homo sapiens* (hMTH1) and MutT gene from *Escherichia coli* (EcMutT). The residues shaded in black indicate identical amino acids. The residues shaded in gray are functionally similar. The red box encloses the residues forming the Nudix motif. Asterisks correspond to the TcMTH catalytic residues, and the hash mark symbol (#) indicates the divalent cation interaction residue.
(TIF)

Table S1 **Normalized amplification of pROCK and MutT parasites nuclear and mithocondrial DNA by QPCR.** Fluorescence values of the long fragments amplification by QPCR normalized with the short fragment amplification. Total DNA from untreated MutT and pROCK epimastigotes cultures were extracted and quantified. Equal amounts of DNA were used to amplify long and short fragments from nuclear and mithocondrial DNA through QPCR protocol. PCR products were quantified by fluorimetric measurement and normalized by short fragment amplification values. 1–4 indicates two biological experiments used to generate two sets of PCR for each target gene. SD = standard deviation.
(DOCX)
