# Supplementary figures and images for: Oxidative Stress and DNA Lesions: The Role of 8-Oxoguanine Lesions in Trypanosoma cruzi Cell Viability
Source: PLoS Negl Trop Dis. 2013 Jun 13;7(6):e2279. doi: 10.1371/journal.pntd.0002279 (PMC3681716; doi:10.1371/journal.pntd.0002279)

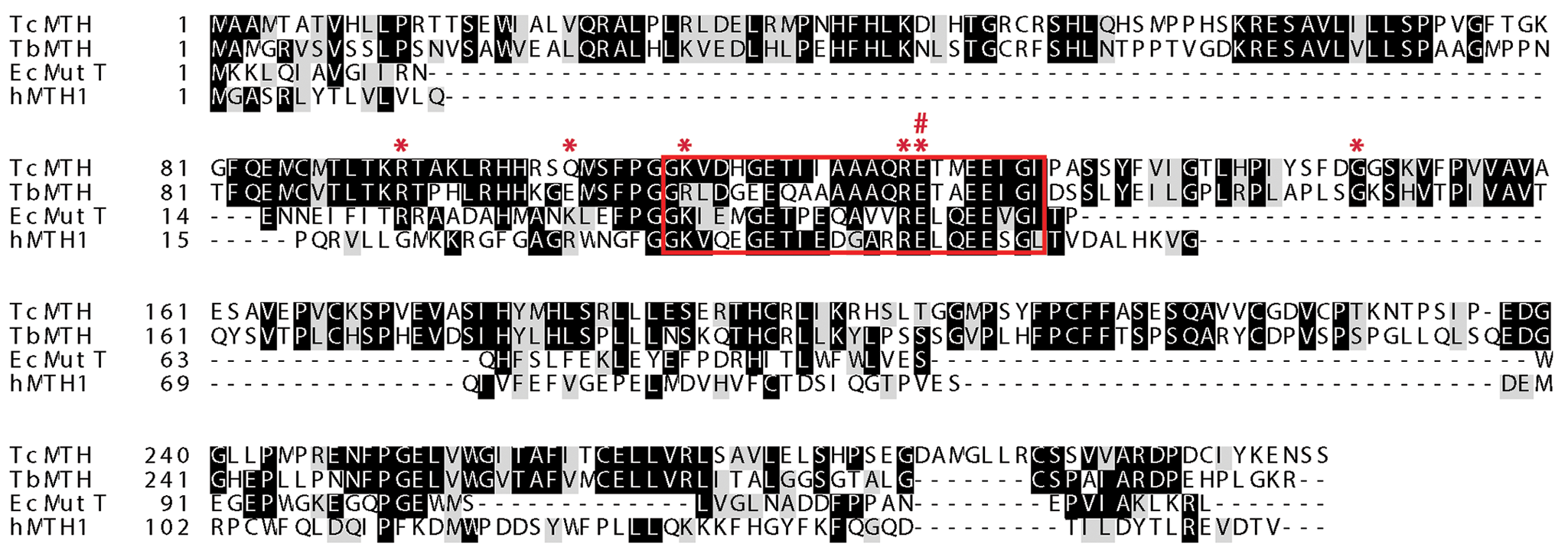

Supplement: Figure S1 — Alignment with the predicted TcMTH product and orthologs. Amino acid sequence comparison of the predicted product of the MTH gene from Trypanosoma cruzi (TcMTH), T. brucei (TbMTH), Homo sapiens (hMTH1) and MutT gene from Escherichia coli (EcMutT). The residues shaded in black indicate identical amino acids. The residues shaded in gray are functionally similar. The red box encloses the residues forming the Nudix motif. Asterisks correspond to the TcMTH catalytic residues, and the hash mark symbol (#) indicates the divalent cation interaction residue. (TIF) [file pntd.0002279.s001.tif]
