# Supplementary material for: Oxidative Stress and DNA Lesions: The Role of 8-Oxoguanine Lesions in Trypanosoma cruzi Cell Viability
Source: PLoS Negl Trop Dis. 2013 Jun 13;7(6):e2279. doi: 10.1371/journal.pntd.0002279 (PMC3681716; doi:10.1371/journal.pntd.0002279)
Supplement: Table S1 — Normalized amplification of pROCK and MutT parasites nuclear and mithocondrial DNA by QPCR. Fluorescence values of the long fragments amplification by QPCR normalized with the short fragment amplification. Total DNA from untreated MutT and pROCK epimastigotes cultures were extracted and quantified. Equal amounts of DNA were used to amplify long and short fragments from nuclear and mithocondrial DNA through QPCR protocol. PCR products were quantified by fluorimetric measurement and normalized by short fragment amplification values. 1–4 indicates two biological experiments used to generate two sets of PCR for each target gene. SD = standard deviation. (DOCX) [file pntd.0002279.s002.docx]

**Supporting information Table 1: Normalized amplification of pROCK and MutT parasites nuclear and mithocondrial DNA by QPCR.**

|  | Nuclear | | Mitochondrial | |
| --- | --- | --- | --- | --- |
|  | MutT | pROCK | MutT | pROCK |
| 1 | 5484.023 | 4053.713 | 34068.65 | 34197.25 |
| 2 | 5492.101 | 3987.111 | 34325.85 | 34238.77 |
| 3 | 5251.268 | 3593.233 | 37994.98 | 29964.98 |
| 4 | 5086.846 | 3473.719 | 38253.52 | 31037.16 |
| Mean | 5328.56 | 3776.944 | 36160.75 | 32359.54 |
| SD | 196.0564 | 286.6276 | 2272.137 | 2190.227 |

Fluorescence values of the long fragments amplification by QPCR normalized with the short fragment amplification. Total DNA from untreated MutT and pROCK epimastigotes cultures were extracted and quantified. Equal amounts of DNA were used to amplify long and short fragments from nuclear and mithocondrial DNA through QPCR protocol. PCR products were quantified by fluorimetric measurement and normalized by short fragment amplification values. 1-4 indicates two biological experiments used to generate two sets of PCR for each target gene. SD = standard deviation.
